# The 1316T>C missenses mutation in MTHFR contributes to MTHFR deficiency by targeting MTHFR to proteasome degradation

**DOI:** 10.18632/aging.202256

**Published:** 2020-12-03

**Authors:** Xi Liu, Yu Li, Menghan Wang, Xiaojun Wang, Limin Zhang, Tao Peng, Wenping Liang, Zhe Wang, Hong Lu

**Affiliations:** 1 Department of Neurology, The First Affiliated Hospital of Zhengzhou University, Zhengzhou 450052, China; 2 The National Clinical Research Center for Geriatric Disease, Xuanwu Hospital, Capital Medical University, Beijing 100053, China; 3 Advanced Innovation Center for Human Brain Protection, Capital Medical University, Beijing 100053, China

**Keywords:** molecular mechanisms, MTHFR deficiency, proteasome degradation, pathogenic mutation, single nucleotide polymophorism

## Abstract

5,10-methylenetetrahydrofolate reductase (MTHFR) deficiency is a rare hereditary disease characterized by defects in folate and homocysteine metabolism. Individuals with inherited *MTHFR* gene mutations have a higher tendency to develop neurodegeneration disease as Alzheimer’ disease and atherosclerosis. MTHFR is a rate-limiting enzyme catalyzing folate production, various SNPs/mutations in the *MTHFR* gene have been correlated to MTHFR deficiency. However, the molecular mechanisms underpinning the pathogenic effects of these SNPs/mutations have not been clearly understood. In the present study, we reported a severe MTHFR deficiency patient with late-onset motor dysfunction and sequenced *MTHFR* gene exons of the family. The patient carries an MD-associating SNP (rs748289202) in one *MTHFR* allele and the rs545086633 SNP with unknown disease relevance in the other. The rs545086633 SNP (p.Leu439Pro) results in an L439P substitution in MTHFR protein, and drastically decreases mutant protein expression by promoting proteasomal degradation. L^439^ in MTHFR is highly conserved in vertebrates. Our study demonstrated that p.Leu439Pro in *MTHFR* is the first mutation causing significant intracellular defects of MTHFR, and rs545086633 should be examined for the in-depth diagnosis and treatment of MD.

## INTRODUCTION

Methylenetetrahydrofolate reductase (MTHFR; EC 1.5.1.20) is a rate-limiting enzyme that plays a central role in folate production and the folate-dependent remethylation of homocysteine [1, 2]. Although congenital MTHFR deficiency (MD) due to genetic mutations is a rare disease [3], it remains to be the most common etiology of congenital folate metabolic dysfunction, which is biochemically characterized by hyperhomocysteinemia, increased plasma cystathionine, and low-plasma methionine [2, 4–6]. Patients with MD manifest a variety of clinical symptoms ranging from early-onset (<1-year-old) diseases such as homocystinuria, severe neurodevelopmental retardation, microcephaly, seizures, and communicating hydrocephalus, to a later onset of psychiatric and gait disturbances, stroke, apnea and neurological deterioration, *etc*, which may lead to coma and even death in the teenage or later in adulthood [7–13].

Human MTHFR belongs to flavoprotein family and is expressed as different isoforms in different tissues/cells [14]. The MTHFR protein consists of an evolutionarily conserved N-terminal catalytic domain (amino acids, aa 1–356) [15] that binds to methylene THF, NADPH, and FAD to catalyze the reducing reaction and a C-terminal regulatory domain (aa 363–656) where the allosteric inhibitor S-adenosylmethionine (AdoMet) binds and regulates the enzyme activity in response to the changes of methionine level in cells [16–18]. The two subunits are connected by a linker region (aa 357–362) [19]. MTHFR plays a central role in folate and homocysteine metabolism. With NAD(P)H as the reducing agent, FAD as the cofactor and NADPH as the electron donor, MTHFR catalyzes the transformation of 5,10-methylenetetrahydrofolate(MTHF) to 5-MTHF that is the major circulating form of folate in the blood and the major methyl donor during the remethylation of homocysteine into methionine [14]. Insufficient MTHFR expression and compromised MTHFR activity under pathological conditions may result in both decreased folate synthesis and elevated homocysteine in blood and urine [5, 20, 21].

The *MTHFR* gene (OMIM: 607093) is located on the short arm of chromosome 1, whose major isoform consists of 12 exons (NM 005957.4, ENST00000376590) [22]. *MTHFR* polymorphisms have been extensively investigated for the correlation with diseases including cardiovascular disease, thrombosis, Alzheimer’s disease, infertility, neural tube defects, cancer, and psychiatric disease, *etc* [23]. To date, 135 disease-associated mutations/SNPs (2019.04 HMGB) have been reported in the *MTHFR* gene [15], and 109 of them identified from 171 families have been correlated with MD [22]. Among these mutations/SNPs, 677C>T (pAla222Val, A222V) and 1298A>C (pGly429Ala, G429A) are the most prevalent variations [24]. The A222V substitution is a genetic risk of mild hyperhomocysteinemia, and in test tubes, it inflicts to the MTHFR protein thermolability (at 46° C) and the loss of FAD interaction on dilution [16, 25], but its relevance to MD remains to be elucidated under physiological conditions. The effects of G429A substitution and other mutations/SNPs are even less understood. Therefore, ACCF/AHA does not recommend the genetic tests of the *MTHFR* gene [26]. Before the functions of these genetic variations are demonstrated, it is more appropriate to refer to them as SNPs that do not imply an adverse effect.

In this study, we report a young patient who manifested severe hyperhomocysteinemia, folate deficiency, cognitive disorder, gait abnormality, scoliosis, and brain white matter abnormalities. *MTHFR* exome sequencing results indicated that the patient inherited from the father the rs748289202 (p.Arg335His, R335H) SNP in the *MTHFR* gene and the rs545086633 (p. Leu439Pro, L439P) SNP in the other allele from the mother. Both of the parents are heterozygous SNP carriers and healthy. Since the rs748289202 has been correlated with MD [13], we, therefore, investigated the effect of rs545086633 and found that L^439^ is highly conserved among vertebrates, and the L439P substitution drastically enhanced proteasomal degradation of MTHFR protein, leading to more than 90% reduction in protein level. To our knowledge, p.Leu439Pro is the first variation in the *MTHFR* gene with strong and clear cut adverse effects intracellularly. It may qualify as an MD pathogenic mutation and should be screened for the cases of MD to determine if there is a combination with another MD associated variation. When either of the parents carries this mutation, the children are at a higher risk of congenital MD, actions should be taken to prevent pre- and postnatal defects. Moreover, as the perturbations of folate and homocysteine metabolism are involved in multiple diseases and aging, animal models with the p.Leu439Pro mutation in *MTHFR* may facilitate the research in these fields and the therapeutic trials of novel medications.

## RESULTS

### Clinical case presentation

**Figure 1:**
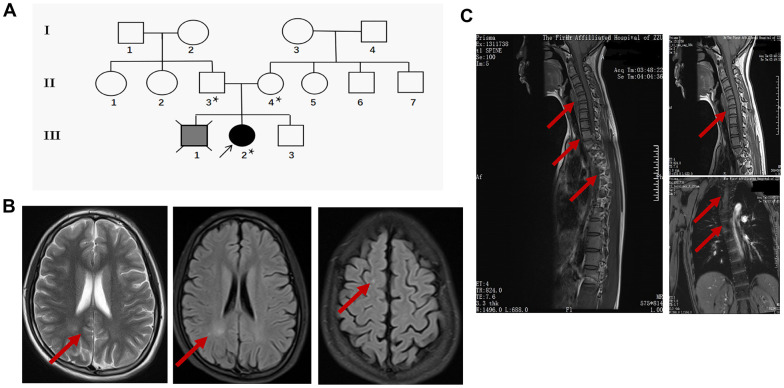
**Clinical data of the proband.** (**A**) Pedigree of the family presenting the p.Leu439Pro mutation. Circles and squares represent females and males, respectively. Clear symbols represent the healthy subject, the grey filled symbol with a cross indicates the deceased subject without clinical diagnosis or genetic examination, and the black filled symbol represents the proband. The asterisk was labeled to the individuals who received the *MTHFR* exon sequencing. (**B**) Representative brain MRI scan images in the basilar ganglia (left and middle panel) and centrum semiovale (right panel) in T2 and FLAIR sequence of the proband. Arrows indicate the white matter injury in the occipital lobe and parietal lobe. (**C**) Representative cervicothoracic combined MRI (left panel) slides of the proband to show the sever reverse gantry of the cervical spine (right upper panel) and scoliosis of the thoracic spine (right lower panel).

The proband (III-2) is a 15 years old female teenager with a chief complaint of “lower limb weakness and walking instability for half a month”. The parents (II-3 age 55, II-4 age 52) of the proband are healthy and had had 3 children. The first child (III-1) without clinical diagnosis or gene test died in his early 20s and suffered congenital mental retardation, and was unable to take care of himself by his death (according to the parents). The second child is the proband. In addition to the chief complaint, she also displayed slow motor response, poor coordination, difficulty in feeding after birth, and a lower intelligence compared with her peers according to her parents. The third child (III-3), a 14 years old male displayed no sign of congenital disorder and has been healthy after birth. None of the three children experienced hypoxia or possible injury at birth. The parent denied a history of drug abuse, pregnancy infection, malformation drug use during pregnancy. They also claimed no similar case in the whole family. The pedigree was made according to the parents’ narration (Figure 1A).

During her first visit, the proband was hospitalized for physical, biochemical, and radiology imaging examinations. The patient showed awake consciousness with normal speech and comprehension. Mildly impaired cognitive disorder in memory and calculation deficits was unveiled by the MMSE scoring (25 points, unable to do the continuity calculation) and MoCA scoring (21 points, unable to draw a clock, visual/special, naming, calculation, abstraction, delayed memory were impaired significantly) test. Physical examination showed that she displayed hypermyotonia and tendon hyperreflexia. Muscle power of the lower extremity was 4 degree for the left and 4+ degree for the right. She could not walk in a straight line and Romberg sign was positive, pathological signs including Babinski sign, Chaddock sign, Rossolimo sign were all positive (Table 1). Electromyography revealed the motor and sensory nerve conduction velocity decreased significantly in the right ulnar nerve and bilateral shin nerve, the deep sensory nerve conduction delayed in the right lower extremity by spinal somatosensory evoked potentials (SSEP). Brain and thoracic spinal cord MRI revealed white matter demyelination of both hemispheres, suggesting white matter disease (Figure 1B). Reverse gantry of the cervical spine and scoliosis of the thoracic spine, which are extremely rare signs in severe MTHFR deficiency cases, were also confirmed by the cervicothoracic combined MRI scan (Figure 1C). Biochemical tests revealed a significant increase in homocysteine and a decrease of folic acid in the blood (Table 2). While the enzyme activity of MTHFR was not tested, the gene tests, physical examination, laboratory test, and MRI scanning of the patient strongly suggested folic and homocysteine metabolism disorder, which could be due to mutations in the *MTHFR* gene.

**Table 1:** Physical examinations of the proband pre- and post-treatment at different time points.

**Physical examination**	**Pre-treatment**	**2weeks**	**3 weeks**	**2 months**	**6 months**
MMSE score (30 points, normal>27)	25 points	—	—	25 points	25 points
MoCA score (30 points, normal>26)	21 points	—	—	21 points	21 points
Muscle tone	hypermyotonia	hypermyotonia	slightly increase	normal	normal
Muscle power(legs) left	4	4	4	4+	5
right	4+	4+	4+	4+	5
Babinski signs	+	+	+	+	+
Chaddock sign	+	+	+	+	+
Rossolimo sign	+	+	+	+	+
Tendon reflex	++++	++++	++++	+++	+++
Romberg sign	+	+	+	+	-
Ataxia	+	+	+	+	-

### Patient benefited from the supplementation of MTHFR enzyme product

During the hospitalization, the patient was prescribed with methionine (50 mg/kg ivgtt, QD), vitamin B12 (5 mg PO,QD), and L-5-MTHF (15 mg PO,QD) to improve the MTHFR deficiency and Baclofen (10mg PO,QD) to alleviate the muscle rigidity. The homocysteine level in the blood decreased gradually after treatment. 2 months after discharge, the patient was followed up for treatment effect. Physical examination revealed that the muscle tone was normal and the power of the legs improved significantly (both sides were 4^+^ degree) despite that the pathological signs were still positive. The patient could walk slowly in a straight line without assistance. However, there was no significant cognitive improvement tested by MMSE and the MoCA scoring. The biochemical values gradually approached to normal standard (Table 2). In the 6-month follow up, the patient could walk freely by herself and the coordination of movements was also normal. There were no apparent side effects of these therapies. The clinical observations suggested that the supplementary treatment alleviated the symptom and further confirmed the methionine insufficiency that could be due to MTHFR deficiency.

**Table 2:** Biochemical result of the patient at different time points.

**Lab test for the blood (Reference value)**	**Pre-treatment**	**Post-treatment**		
		2 weeks	3weeks	2months
Homocysteine (0-15μmol/L)	122.6	63.35	54.73	49.49
Folic acid (5.21-20ng/mL)	2.54	-	-	>25.60
Vitamin B12 (200-1100pg/mL)	682.00	-	-	2000.00
APTT (26-40s)	25.90	-	-	32.80
Fibrinogen (2-4g/L)	1.92	-	-	2.24

### The MTHFR exon sequencing revealed two rare non-synonym SNPs in the MTHFR gene

**Figure 2:**
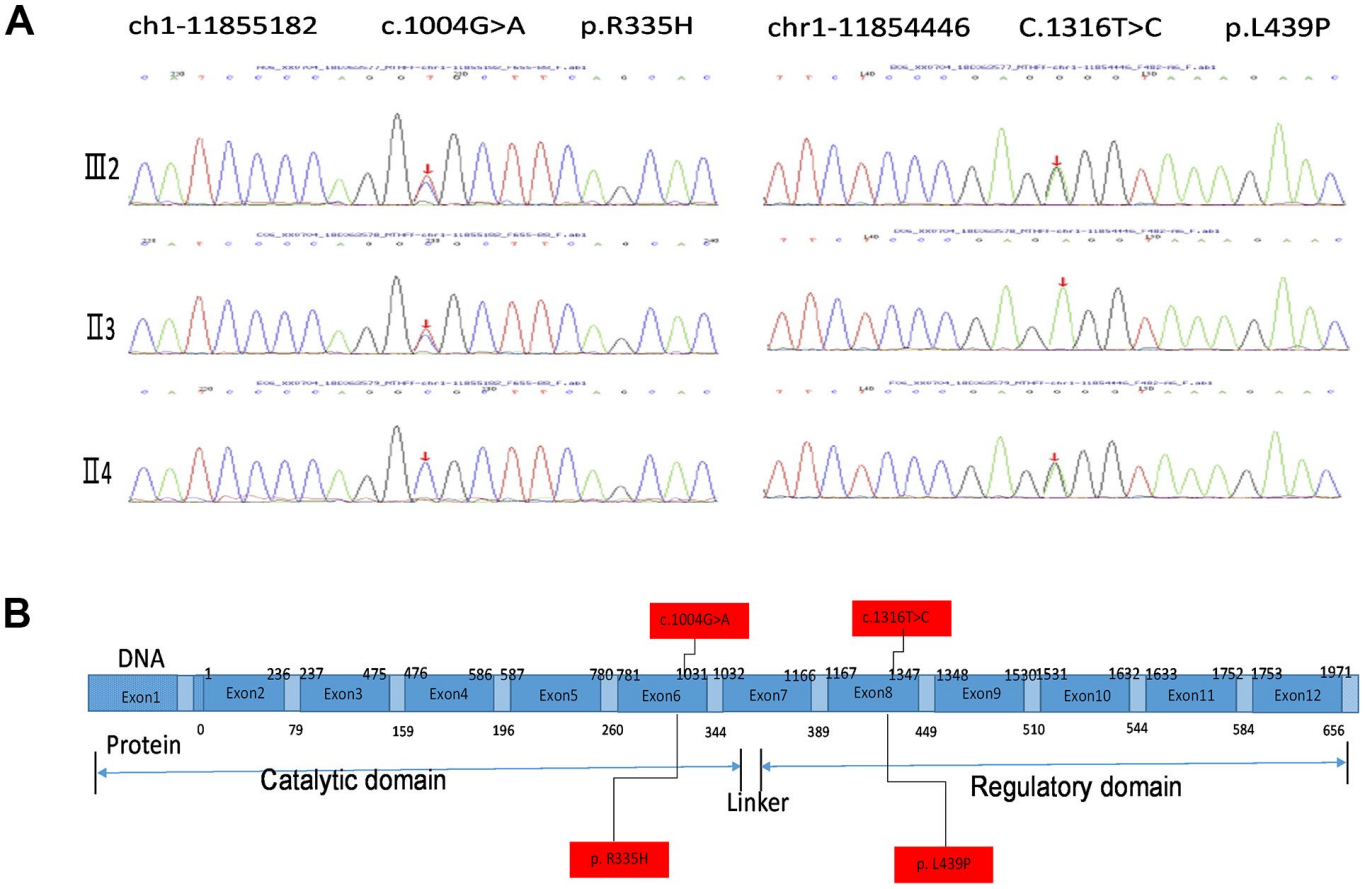
**MTHFR exosmic sequencing results of the proband and the parents.** (**A**) MTHFR exosmic sequencing indicates a combined heterozygotic mutation in the proband (III2), the disease-associated mutations 1004G>A(R335H) heredities from the proband’s father (II3) and the p.Leu439Pro (L439P) mutation inherited from her mother (II4). (**B**) Schematic chart depicting the structure of the *MTHRF* gene and the protein. The letters highlighted with red box indicate the SNP/mutation sites in the gene and the corresponding amino acid residuals.

Exon sequencing of the *MTHFR* gene was ordered to make a genetic diagnosis. Surprisingly, the sequencing identified very rare compound heterozygous mutations of the proband’s *MTHFR* gene (Figure 2A). The *MTHFR* 1004G>A(R335H) that has been reported to be disease-associating was inherited from the proband’s father, and the *MTHFR* 1316T>C(L439P) SNP with unknown effects was from the proband’s mother as depicted in the schematic chart in Figure 2B. Both of the parents are heterozygous SNP carriers with one allele containing a SNP, and the other allele containing no rare SNPs. Of note, L^439^ in MTHFR protein is highly conserved among vertebrates (Table 3), and the isoleucine (I) at this position in chicken and zebrafish is usually interchangeable with leucine (L). Thus, the L^439^ within the regulatory domain site may play an indispensable role in MTHFR functions.

**Table 3:** MTFR p.Leu439Pro conservation in paralogs.

**Species**	**Sequence ID**	p.L439*
Homo sapiens	NP_001317287.1	GEELTSEESVFEVFVLYLSGEPNRNGHKV
Gorilla	XP_018866767.1	GEELTSEESVFEVFVLYLSGEPNRNGHKV
Mus musculus	AAD20313.1	GEELTSEESVFEVFEHYLSGEPNRHGYRV
Oryctolagus cuniculus (Rabbit)	XP_008273608.1	GEELTSEESVFEVFVRYLSGEANQQGYKV
Gallus gallus (Chicken)	NP_001315420.1	GEELTGEESVFEVFTCYITGEPNKNGHRV
Danio rerio (Zebra fish)	AFB35148.1	GEELMSEESVYEVFTNYITGQTNRSGHKV

Sequences were aligned with BLAST and the position of L439 in human MTHFR is highlighted in red. Accession numbers of human MTHFR and its orthologs are provided.

### The L439P MTHFR substitution facilitated MTHFR degradation via the proteasomal pathway

**Figure 3:**
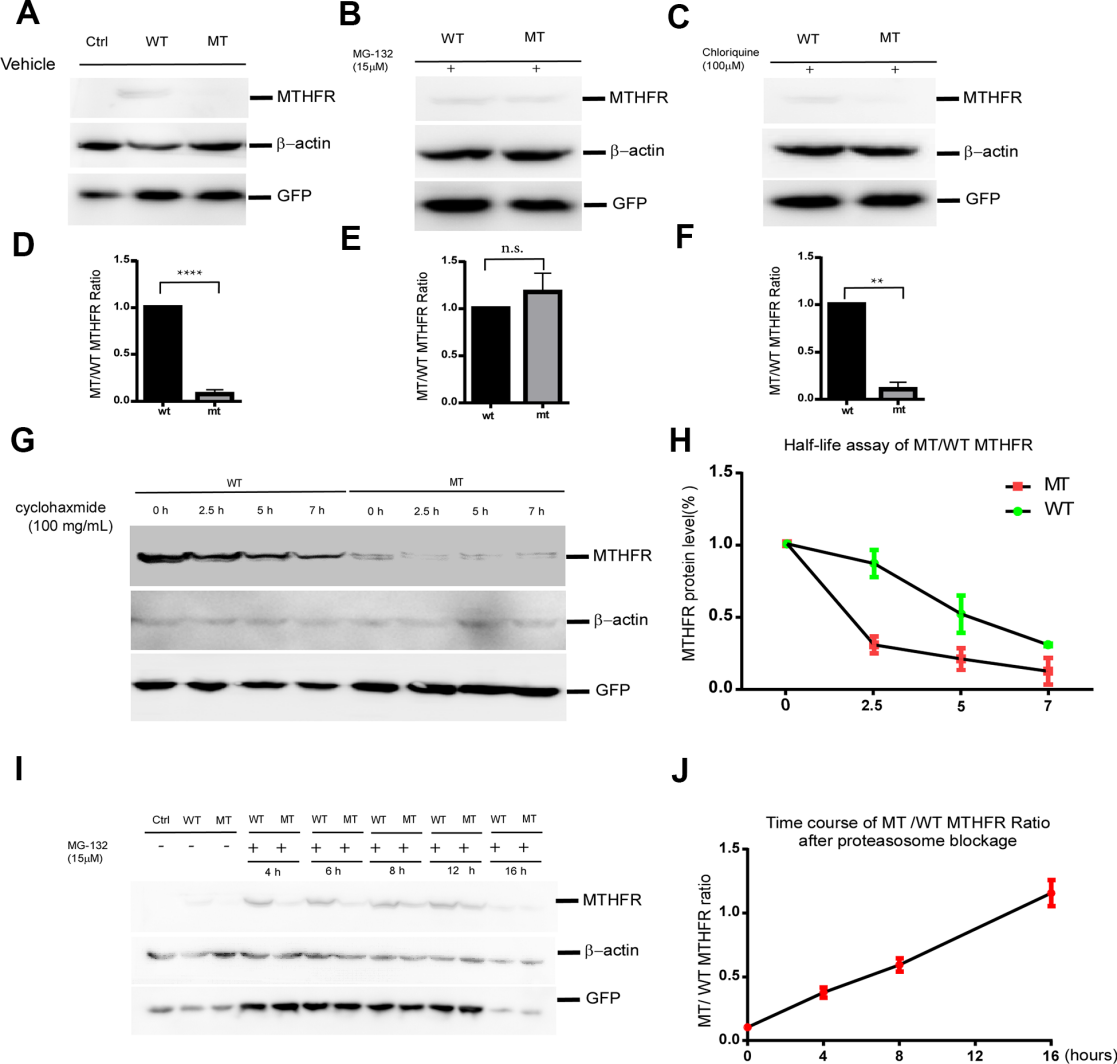
**The p.Leu439Pro mutation in MTHFR facilitated MTHFR degradation via the proteasomal pathway.** Western blot bands (**A**) and the quantification (**D**) demonstrated that at equal transfection efficiency (as indicated by GFP expression), the protein level of over-expressed MTHFR_L439C_ was significantly lower than that of MTHFR_WT_ (*p*<0.0001, n=3 replicates).Lysosome blockage by chloroquine failed to elevate the mutant MTHFR protein level (**C**, **F**. *p*=0.1746), whereas proteasome blockage by MG132 significantly increased MTHFR_L439P_, and abolished the difference between the wild type and mutant MTHFR (**B**, **E**. *p*<0.01, n=3 replicates). (**G,** **H**) Half-life assay of the WT and MTHFR_L439P_ by using cycloheximide to block protein synthesis and chase the remaining protein level by western blot at 0, 2.5, 5, 7 hours later(**G**). The half-life of WT MTHFR is around 5 hours whereas the MTHFR_L439P_ was almost depleted within 2.5 hours (H, n=3). (**I**, **J**) Representative Western blot result of WT and the mutant MTHFR protein expression on a time course of MG132 treatment(I), and quantifications (**J**), (n=2 replicates). All data are expressed as Mean±SEM. ****p<0.0001, **p<0.01 (by Student's *t*-test).

To investigate the effect of L439P substitution, an equal amount of plasmids expressing wild type and mutant MTHFR were transfected into HEK293 cells. pEGFP-N2 was co-transfected with MTHFR plasmids as an internal control for equal transfection efficiency. Western blot demonstrated that the protein level of MTHFR_L439P_ was only 7.30±2.39% (*p*<0.0001, n=3) that of wild type MTHFR_WT_, whereas the co-expressed GFP showed no difference between the two groups (Figure 3A–3D). As the cDNA expressions of MTHFR (WT and mutant) were both driven by the artificial CMV promoter, and unlikely involves RNA splicing, this result strongly suggests that the L439P substitution may accelerate MTHFR degradation.

Protein half-life assay using cycloheximide (CHX) to blocking protein synthesis revealed that the half-life of wild-type MTHFR was around 5hr. By contrast, the L439P mutant of MTHFR was almost depleted within 2.5hr after the addition of CHX(Figure 3G, 3H). The shorter half-life of the MTHFR_L439P_ suggested that this mutant underwent faster degradation than the wild-type MTHFR.

To identify the degradation pathway through which MTHFR_L429P_ is degraded, HEK293 cells expressing either MTHFR_WT_ or MTHFR_L439P_ were treated with DMSO as solvent control, proteasome inhibitor MG132, or lysosome inhibitor chloroquine (CHL) for 12 hours. While CHL failed to elevate the mutant MTHFR protein level (Figure 3C, F. *p*=0.1746 n=3), MG132 almost abolished the difference between the wild type and the mutant MTHFR (Figure 3B–3E. *p*=0.0023 n=3). Hence, the mutant MTHFR protein is robustly degraded by the proteasome after synthesis.

Interestingly, although within the initial 8 hours of MG132 treatment, mutant MTHFR was quickly upregulated and approaching the level of wild type MTHFR, prolonged MG132 treatment to 16 hours reduced wild type MTHFR more than it did to the mutant, which brought wild type and mutant MTHFR at a similar level (Figure 3I, 3J). At this time point of MG132 treatment, the co-transfection control GFP was reduced as well, therefore, the reduction of wild type MTHFR is unlikely a specific effect of proteasome inhibition.

## DISCUSSION

In the present study, we reported a novel mutant form of MTHFR from a Chinese Han family. The proband exhibited typical clinical presentation of mild cognitive disorder, legs powerless, gait disorder, severe scoliosis, electrophysiology abnormality, and biochemically displayed severe hyperhomocysteinemia, folate deficiency. MRI scanning also detected brain white matter abnormalities. All of these symptoms and tests suggested MD of this patient. Exome sequencing of the *MTHFR* gene revealed that the patient received a disease-associated SNP from the father and an uncharacterized SNP from the mother. Our data demonstrated that the uncharacterized SNP is probably deleterious as well because its effect on MTHFR expression was next to gene knockout *in vitro*. Unlike her healthy parents who have a normal *MTHFR* allele to compensate MTHFR functions, the proband patient does not have such a rescuing mechanism and therefore suffered congenital MD. Hence, both of the SNPs/mutations from the parents cause diseases in an autosomal recessive manner. In a similar case, a newborn baby carrying compound mutations in the *MTHFR* gene (c.523G>A in one allele, and c.1166G>A in the other) had extremely low MTHFR enzyme activity and died six weeks after birth. By contrast, both of the parents presented no neurological symptoms [27]

At this stage, we cannot rule out other recessive mutations in the patient that are involved in pathogenesis or symptom aggravation, because only the *MTHFR* gene was sequenced. However, as methionine supplement improved all the symptoms except cognitive impairment and bone deformity as spinal cord scoliosis that reflect a defect during early development, other disease-associating mutations, if any, are probably in the same pathway of methionine production.

While hundreds of *MTHFR* SNPs correlating to MTHFR deficiency have been identified, their functions under physiological conditions remain unknown. The p.Ala222Val substitution is hitherto the best characterized *MTHFR* gene variation, yet, which function of MTHFR is blunted in cells by this substitution is still a question mark. Based on the clinical manifestations, examinations and the efficacy of therapies, we first confirmed MD in the proband, which is probably due to MTHFR genetic mutations. Exome sequencing of the *MTHFR* gene identified two rare SNPs inherited from both parents, respectively. Our cell-based experiments indicated that the L439P substitution (rs545086633) almost abolished MTHFR expression by enhancing MTHFR degradation via the proteasome. All of genotyping, function analysis, clinical diagnosis, and the effect of therapy leads to the conclusion that rs545086633 is a detrimental mutation instead of a benign SNP. Since rs545086633’s effect on MTHFR is next to gene knockout and the mother containing this mutation is healthy, one allele of wild type *MTHFR* in human is sufficient to fulfill the functions at the basal level, but at the time of higher requirement of MTHFR activity, or when MTHFR activity is decreasing, such as during aging, both alleles might become necessary to prevent MTHFR insufficiency.

Our results suggested that gene examination for the p.LeuL439Pro mutation could be performed for MD cases and people susceptible to MD. When either of the parents carries the p.LeuL439Pro mutation, a prenatal examination of the *MTHFR* gene may benefit the prognosis of congenital MD. An early methionine and folate supplementation could minimize the defects by congenital MD during early development. In summary, we reported a severe MTHFR deficiency patient carrying in the *MTHFR* gene a disease-associating SNP in one allele, and the rs545086633 SNP with unknown disease relevance in the other. The rs545086633 SNP (p.Leu439Pro) results in an amino acid substitution in a highly conserved position L^439^ in MTHFR, and drastically decreases mutant protein expression by promoting proteasomal degradation. Thus, p.Leu439Pro qualifies as a pathogenic mutation.

Gene mutations may impose a high risk of diseases. They could be resulted from exogenous damage and the failure of internal polymerase repair [28], and passed on from the ancestors to the descendants. Mutations causing amino acid substitution may impair protein structures, stability, and functions [29]. To the best of our knowledge, we identified that p.Leu439Pro is the first variation in the MTHFR gene with strong and clear cut adverse effects intracellularly. It could be screened for the cases of MD. When either of the parents carries this mutation, the children are at higher risk of congenital MD, actions should be taken to prevent developmental and postnatal defects. Moreover, animal models with the p.Leu439Pro mutation in MTHFR may facilitate the research in the fields of folate and homocysteine metabolism and the therapeutic trials of novel medications.

## MATERIALS AND METHODS

### Plasmids construction

The pENTER-MTHFR_wt_ plasmid expressing human MTHFR (accession No.: XP_011539797.1) was purchased from Vigene Bioscience (Shandong, China). cDNA of MTHFR was PCR amplified and inserted in pcDNA4-myc-His A vector. L439P substitution was introduced into wild type MTHFR by PCR mutagenesis. All plasmid was confirmed by sequencing.

### Cell culture, transfection and drug treatment

Human embryonic kidney cells (HEK293) line was maintained in high-glucose Dulbecco’s modified Eagle’s medium (DMEM) supplemented with 10% fetal bovine serum and 100 U/mL penicillin–streptomycin. For protein expression, cells were transfected with equal amount of pcDNA4-MTHFR_wt_, the pcDNA4- MTHFR^mut^ plasmid and pcDNA4 as vector control with Lipofectamine 2000 reagent (Invitrogen, San Diego, CA, USA). GFP was coexpressed with MTHFR variants as equal transfection control. The transfected cells were treated with autophagy-lysosome pathway inhibitor chloroquine (Sigma–Aldrich, St. Louis, MO, USA) at 100μM or ubiquitin-proteasome pathway inhibitor MG132 (Calbiochem, San Diego, CA, USA) at 10μM for indicated time before harvest. For half-life assay, equal amount of HEK293 cells transfect with equal amount of pcDNA4-MTHFR_wt_, the pcDNA4-MTHFR^mut^ plasmid. pEGFP-N2 was co-transfected with MTHFR plasmids as a marker for equal transfection efficiency. The cells were harvested for western blot at 0, 2.5, 5, 7 hours later after treated with 100mg/mL cycloheximide.

### Western blot analysis

Cells were harvested with ice-cold PBS and lysed in RIPA buffer (TrisHCl, 50 mM; NaCl, 150 mM; Triton X-100, 1%; deoxycholate, 1%; and SDS, 0.1%; supplemented with protease inhibitors). Protein concentration was determined with the Bradford protein assay. Cell lysates were separated on 7% Tris-glycine SDS-PAGE gels and transferred onto PVDF membranes. The wild-type or the mutant MTHFR were detected by rabbit anti-6×His antibody (Affinity, China) at 1:1000 dilution. The HRP-conjugated goat anti-rabbit IgG was diluted at 1:5000 (Shenggong, shanghai, China) was use for band visualization. For protein expression analysis, all bands were normalized by the bands intensity of the expression of β-actin.

### Data analysis

Western blot analysis was performed with 3 independent experiments. All data are expressed as mean±SEM, unpaired students’ *t*-test followed by Welch’s correction was used for comparison of the difference between the two groups. *P*<0.05 was considered to be statistically different. Prism Graph pad 6.0 (San Diego, CA, USA) was used to process the data.
